# The complete chloroplast genome of *Euphorbia hirta* (Euphorbiaceae), a commonly used medicinal plant in China

**DOI:** 10.1080/23802359.2021.1945506

**Published:** 2021-06-28

**Authors:** Yancheng Zhang, Zhaocen Lu, Deng Zhang, Jingjian Li

**Affiliations:** a College of Pharmacy, Guilin Medical University, Guilin, PR China; b Guangxi Institute of Botany, The Chinese Academy of Sciences, Guilin, PR China

**Keywords:** Chloroplast genome, *Euphorbia hirta*, phylogenetic analysis

## Abstract

Plants in the genus *Euphorbia* have been widely used as herbal medicine, and for ornamental horticulture and biofuel production. In this study, we report the complete chloroplast genome of *Euphorbia hirta* which is known as the ‘asthma-plant’ due to its medicinal use. The chloroplast genome of this species is 164,340 bp in length, including a pair of inverted repeat regions (IRs) (27,354 bp) that are divided by a large single-copy region (LSC) (91,373 bp) and a small single-copy region (SSC) (18,259 bp). The chloroplast genome of *E. hirta* contains 111 unique genes (77 protein-coding, 30 *tRNA*, and four *rRNA*), 19 of which are duplicated in the IR regions. The overall GC content is 35.4%. Phylogenetic analysis fully resolved *E. hirta* groups with other species of *Euphorbia*. The complete chloroplast genome of *E. hirta* provides useful information that can be used to distinguish related species and reconstruct evolutionary relationships.

*Euphorbia hirta* L. is classified in the Euphorbiaceae, and is a small, annual herb originating in the Americas and now established as a pantropical weed (Nyeem et al. 2017). This plant is a very popular herb among practitioners of traditional Chinese medicine and is widely used as a decoction or infusion to treat various ailments including diarrhea, heartburn, asthma, bronchitis, and intestinal parasites (Kumar et al. 2010). Modern pharmacological research has demonstrated that *E. hirta* has various pharmacological activities, such as anti-anaphylaxis, anti-cancer, and anti-inflammation (Bhagwat et al. 2008). In addition to ethnomedicinal research, some studies also have described the evolution and phylogeny of *E. hirta* (Yang et al. 2012; Horn et al. 2014). However, as a member of the C4 clade of *Euphorbia*, little is known about the molecular structure and composition of its chloroplast genome, which might have some bearing on its photosynthetic system. In recent years, the rapid progress of next-generation sequencing has enabled the efficient analysis of chloroplast genomes (Zha et al. 2020). In the genus *Euphorbia*, the chloroplast genome of some species, such as the biofuel plant *E. tirucalli* and medicinal plant *E. lathyris* have been reported (Zhang et al. 2019; Ma et al. 2020); however, the chloroplast genome of *E. hirta* has not yet been deciphered. Here, we assembled and analyzed the complete chloroplast genome sequence of *E. hirta* to contribute to its systematics, evolutionary history and serve as a resource for future genetic studies.

Fresh young leaves from *E. hirta* were collected at the Guangxi Institute of Botany, The Chinese Academy of Sciences, Guilin, China (latitude: 25.0677; longitude: 110.3037). The voucher specimen was deposited at the herbarium of Guangxi Institute of Botany (contact person: Zhaocen Lu, email: 474947278@qq.com) under the voucher number IBK-450330180910046LY). Total DNA was extracted using the DNeasy Plant Mini Kit (QIAGEN, Hilden, Germany). The quality and concentration of the DNA product were assessed using agarose gel electrophoresis and an Agilent Bioanalyzer 2100 (Agilent Technologies, Santa Clara, CA). The DNA library with an insert size of 270 bp was constructed and sequenced on an Illumina HiSeq2000 platform using paired-end read lengths of 150 bp. The raw reads were assembled into whole chloroplast genome in a multi-step approach using the program GetOrganelle (Jin et al. 2020). Briefly, a total of 7,522,764 raw reads were aligned with the reference genome of *E. tirucalli* (MH890571). As a result, 3,779,882 reads were mapped to the reference genome and then de novo assembled using SPAdes version 3.6.0 (Bankevich et al. 2012). The assembled chloroplast genome was annotated by the combination of CPGAVAS2 (Shi et al. 2019) and GeSeq (Tillich et al. 2017). Where necessary, start and stop codons, and boundaries between exons and introns were adjusted manually.

The assembled chloroplast genome of *E. hirta* is 164,340 bp in length with a typical quadripartite structure which includes a pair of short inverted repeat regions (IRa and IRb) consisting of 18,259 bp each, a large single-copy (LSC) region of 91,373 bp, and a small single-copy (SSC) sequence of 18,259 bp. The chloroplast genome contains a total of 111 genes, including 77 protein-coding, 30 *tRNA* and four *rRNA*. The gene content and gene order are similar to the chloroplast genomes of other species in the Euphorbiaceae (Khan et al. 2020). Fifty-eight protein-coding genes and 22 *tRNA* genes are located in the LSC region. Eleven protein-coding genes and one tRNA are located in the SSC region. Eight protein coding, 7 *tRNA* and 4 *rRNA* genes are duplicated in the IRa and IRb regions. The overall GC content of the *E. hirta* chloroplast genome is 35.4%, which is similar with those of other member of Euphorbiaceae and angiosperms (Qian et al. 2013; Khan et al. 2020).

To confirm the phylogenetic position of *E. hirta*, a phylogenomic analysis was performed based on 16 published species within the Euphorbiaceae and two basal species (*Pyracantha fortuneana* and *Linum usitatissimum*) from other families. A total of 77 protein-coding genes shared by all species were extracted and were aligned using MUSCLE (Edgar 2004). The maximum likelihood (ML) analysis was conducted using RAxML version 8.0 software (Stamatakis 2014) based on the GTR + GAMMA nucleotide substitution model with 1000 bootstrap replicates. For phylogenetic inferences, *P. fortuneana* (Rosaceae) was designated as the outgroup. The ML tree indicated that *E. hirta* and *E. maculata* cluster as a sister group in *Euphorbia*, and their classification as members of *Euphorbia* in the subgenus Chamaesyce (Figure 1) is consistent with previous studies (Yang et al. 2012; Khan et al. 2020; Ma et al. 2020). The chloroplast genome of *E. hirta* contributes to the growing number of chloroplast genomes for phylogenetic and evolutionary studies in the genus *Euphorbia*.

**Figure 1.**
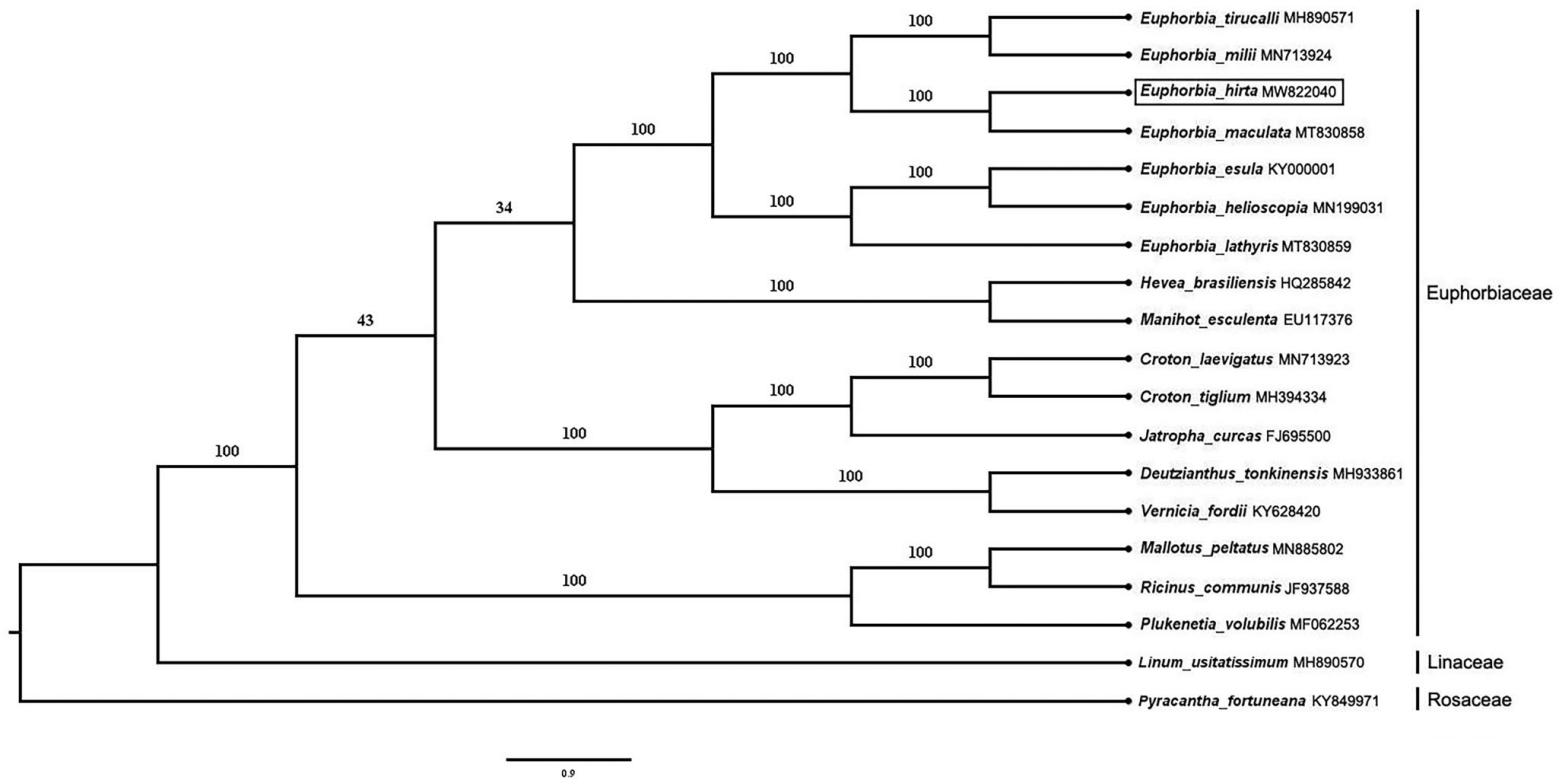
Maximum likelihood phylogenomic tree inferred with RAxML for *E. hirta* using 77 protein-coding genes from complete chloroplast genomes. *P. fortuneana* used as out-group species. Bootstrap support values based on 1000 replicates are given at the nodes.

## Data Availability

The genome sequence data that support the findings of this study are openly available in GenBank of NCBI at (https://www.ncbi.nlm.nih.gov/) under the accession no. MW822040. The associated BioProject, SRA, and Bio-Sample numbers are PRJNA721856, SUB9483761, and SAMN18740319, respectively. Tree files of 19 species and genes for phylogenetic analysis were deposited at Figshare: https://doi.org/10.6084/m9.figshare.14420648.
